# Medical and Psychiatric Consultation for Patients Considering State-Regulated Psilocybin Services: A Risk-Reduction Model and Two Illustrative Cases

**DOI:** 10.21203/rs.3.rs-10779254/v1

**Published:** 2026-08-25

**Authors:** MICHAEL SARVI, Aryan Sarparast

**Affiliations:** Oregon Health & Science University; Oregon Health & Science University

**Keywords:** psilocybin, Oregon Psilocybin Services, state-regulated psychedelic services, risk assessment, risk reduction, alcohol use disorder, major depressive disorder

## Abstract

**Background:**

State-regulated psilocybin services have expanded access outside research settings. Patients seeking these services may have medical and psychiatric complexity not represented in clinical trials, while guidance for clinicians advising them remains limited. We introduce the Psilocybin Education and Assessment Collaborative for Excellence (PEACE), a consultation model within a traditional healthcare system developed to support patients considering Oregon Psilocybin Services (OPS).

**Methods:**

We describe the PEACE model’s structure and clinical approach, including individualized assessment, evidence appraisal, education, risk-reduction planning, and care coordination. We present two longitudinal cases illustrating clinical complexity and outcomes after accessing OPS.

**Results:**

The first case involves a middle-aged man with alcohol use disorder who experienced early improvement after two OPS sessions and ultimately achieved abstinence through a nonlinear recovery process. During abstinence, he underwent a distressing high-dose unsupervised psilocybin experience, after which he received PEACE-supported integration care. The second case involves an older woman with major depressive disorder who completed supervised antidepressant tapers and underwent two supervised psilocybin experiences. Her depression worsened three months after the second experience, and she returned to PEACE for medication and integration support.

**Conclusion:**

These cases illustrate the challenges of extrapolating psychedelic clinical trial findings to real-world settings and the importance of longitudinal follow-up, as early improvement may not reflect longer-term, nonlinear recovery trajectories. Consultation models such as PEACE may support informed decision-making, risk reduction, continuity of care, and integration of psilocybin-related care into existing healthcare systems.

## Introduction

In 2020, Oregon became the first state to establish a regulated framework for supervised psilocybin use through the Oregon Psilocybin Services Act.^1^ Colorado and New Mexico subsequently enacted distinct regulated-access frameworks in 2022 and 2025, respectively.^2,3^ Between January 1 and December 31, 2025, Oregon service centers reported serving 5,935 clients, who sought psilocybin services for diverse reasons, including health and wellness, personal growth, and self-reported mental health concerns such as anxiety (23.8%), depression (22.0%), and posttraumatic stress disorder (13.1%).^4^ As many individuals seek state-regulated psilocybin services to address mental health concerns, questions arise about how these services should interface with existing healthcare systems, particularly when clients have co-occurring psychiatric diagnoses or medical conditions. These questions also extend across state lines: 32.6% of Oregon clients in 2025 were nonresidents.^4^

Alongside expanding state-regulated access, clinical research on psilocybin has increased substantially in the last two decades.^5^ Randomized placebo-controlled trials have evaluated psilocybin for depression, cancer-related psychological distress, and alcohol use disorder.^6^ However, in a recent scoping review, randomized trials accounted for only 12% of included studies, and the median sample size across primary studies was 22 participants.^6^ The broader evidence base also remains limited by restrictive eligibility criteria, short follow-up periods, challenges with blinding, and potential conflicts of interest.^6,7^ Despite recent advances, including sponsor-reported positive Phase 3 results for a proprietary psilocybin formulation in treatment-resistant depression,^8^ psilocybin remains a Schedule I substance with no FDA-approved indications.^9,10^

Amid growing public interest, limited guidance exists regarding patient selection, potential contraindications, medication management, and risk stratification for medically and psychiatrically complex patients in routine clinical practice.^11,12^ Oregon’s model has expanded access to psilocybin within a structured framework that includes preparation, supervised administration, and optional integration with trained, licensed facilitators.^13^ While this framework may meet the needs of many clients, individuals with greater medical or psychiatric complexity may benefit from additional clinical assessment and guidance.

We describe the Psilocybin Education and Assessment Collaborative for Excellence (PEACE), a clinical consultation service designed to complement Oregon Psilocybin Services (OPS) by providing medical and psychiatric risk assessment, education, and risk-reduction guidance. We illustrate the application of this model through two longitudinal cases: a man with alcohol use disorder whose recovery followed a nonlinear course after psilocybin use and a woman with recurrent major depressive disorder who experienced initial symptom relief but later required extensive aftercare during a severe depressive relapse.

### The PEACE Model of Care

Founded in 2023 within an academic medical center in Oregon, the Psilocybin Education and Assessment Collaborative for Excellence (PEACE) functions as a psilocybin consultation service. PEACE receives referrals from primary care, psychiatry, oncology, and palliative care clinicians, as well as self-referrals. Consultations focus on individualized medical and psychiatric risk assessment, education, risk reduction, and recommendations regarding aftercare when indicated. PEACE consultants neither supply psilocybin nor facilitate dosing sessions.

**Figure 1:**
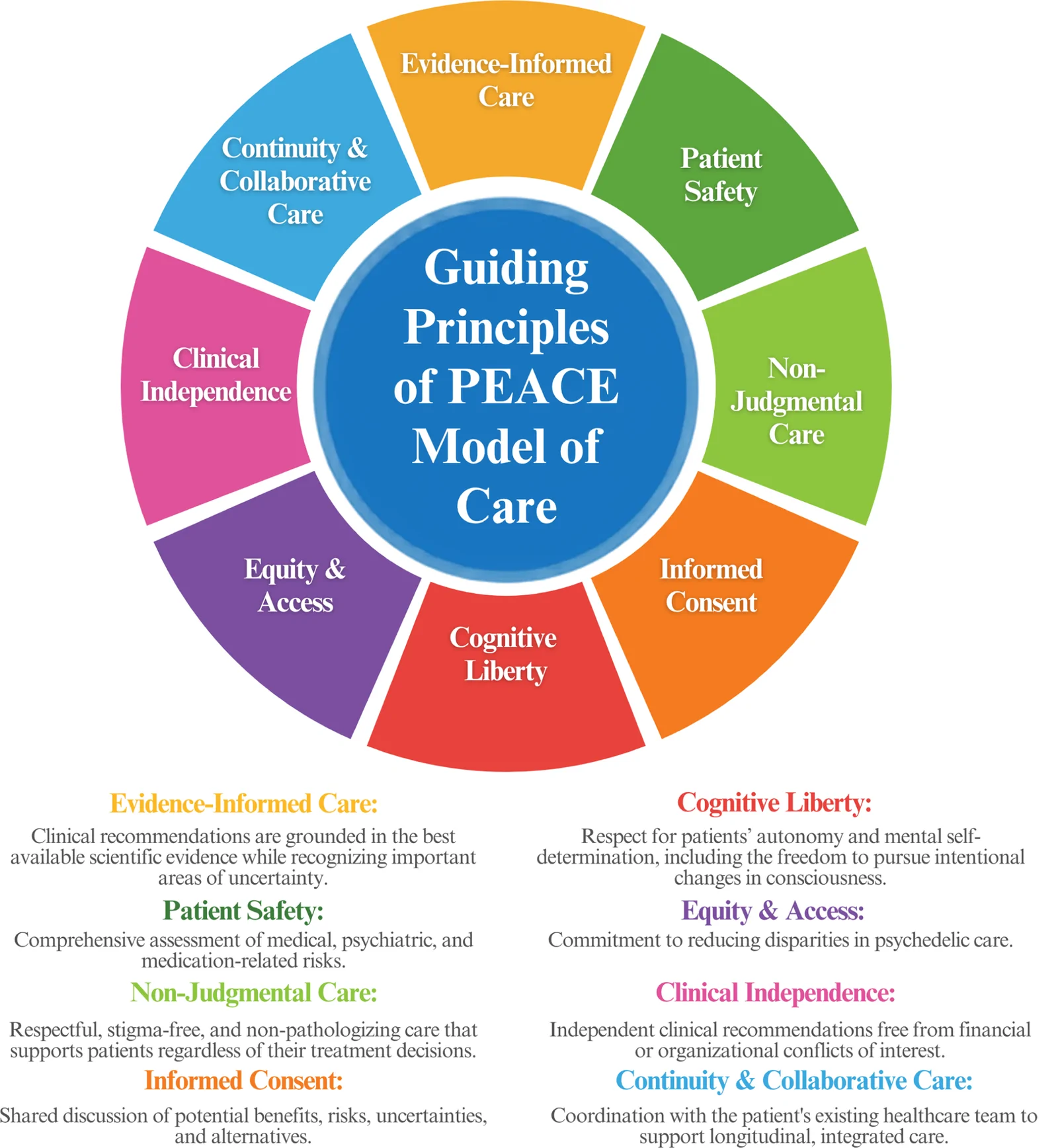
Guiding Principles of PEACE Model of Care

Consultations are conducted by clinicians with specialized training in psychedelic research and risk reduction, including a psychiatrist and a family medicine physician, and are guided by core clinical and ethical principles that inform the PEACE model of care (Fig. 1). When clinically indicated, PEACE may collaborate with referring clinicians and other mental health professionals within the institution. Patients typically complete two approximately 90-minute visits: an Assessment Visit and an Education Visit. Additional visits are available for aftercare or further consultation when needed. Consultations are billed to insurance, including Medicare and Medicaid.

The first visit, termed the Assessment Visit, involves a comprehensive biopsychosocial assessment, review of relevant health records and diagnostic studies, and appraisal of the evidence for psilocybin use in the patient’s clinical context. Core assessment domains are summarized in Table 1. When appropriate, psychedelic preparedness is assessed using the Psychedelic Preparedness Scale to identify potential areas for further education.^14,15^ At the conclusion of the visit, the consultant shares a preliminary clinical synthesis, including the principal anticipated medical and psychiatric safety concerns and areas requiring further review.

During the second visit, termed the Education Visit, the consultant provides a more detailed, individualized synthesis of the patient’s medical and psychiatric risk profile. This synthesis, together with findings from the Psychedelic Preparedness Scale when administered, guides education, medication recommendations, care coordination, and risk-reduction planning. Educational topics commonly include medication interactions; expected subjective and physiological effects, including psychological distress or trauma re-emergence; and the importance of social support. Particular emphasis is placed on intention setting, establishing rapport with a qualified facilitator or therapist, discussing boundaries and consent regarding touch during the dosing session, and arranging psychotherapy or counseling to support preparation beforehand and integration afterward.

### Clinical Complexity and Safety Considerations in PEACE Consultations

PEACE encounters individuals with medical and psychiatric complexity that may increase the risks associated with psilocybin use or limit the applicability of existing evidence. Many would likely be excluded from contemporary clinical trials because of psychiatric risk, medical comorbidity, medication-related concerns, or limited safety data in their clinical populations. Common clinical trial exclusion criteria relevant to these consultations are summarized in Tables 2 and 3. Such individuals require individualized risk assessment, transparent communication of uncertainty, risk-reduction guidance, and coordination with existing treatment teams.

In a retrospective study of the first 29 patients evaluated through PEACE, the most common consult-relevant psychiatric diagnoses were unipolar depression (55.2%), posttraumatic stress disorder (41.4%), and anxiety disorders (34.5%); 86.2% also reported lifetime trauma exposure.^14^

Beyond these common presentations, psychiatric concerns encountered in PEACE consultations have included bipolar spectrum disorders, first-degree family histories of bipolar or psychotic disorders, remote substance-induced psychosis or delirium, elevated suicide risk, borderline personality disorder, active substance use disorders, and complex psychotropic medication regimens. Consultants strongly discourage psilocybin use in circumstances such as acute suicidality, recent mania or psychosis, recent psychiatric hospitalization, or impaired decision-making capacity. For individuals with conditions commonly excluded from contemporary psilocybin trials (Table 2), consultants discuss the limited applicability of existing evidence and resulting uncertainty regarding risk. Where applicable, proposed risk-stratification frameworks, such as that described by Downey et al. for family history of bipolar disorder, may help structure these discussions.^16^ Medication assessment includes potential interactions, risks of medication changes or antidepressant tapering, and particular caution with lithium given reports of seizures with concurrent classic psychedelic use.^17^

Medical concerns have included seizure disorders, intracranial masses, prior stroke, Parkinson’s disease, multiple sclerosis, type 1 diabetes managed with an insulin pump, severe cardiovascular disease, difficult-to-control hypertension, and advanced malignancy. Complexity has been particularly common among individuals with advanced cancer or progressive neurologic disease and may require coordination with relevant specialists and existing care teams, including oncology, neurology, psychiatry, palliative care, and primary care. When psilocybin is considered high risk or supporting evidence is limited, clinicians may discuss alternatives aligned with the patient’s goals, including psychopharmacology, psychotherapy, neuromodulation, ketamine-assisted psychotherapy, breathwork, and contemplative or spiritual practices. Patients who elect to proceed may receive additional safety-planning guidance, including consideration of facilitators with healthcare backgrounds. In end-of-life contexts, prognosis and goals of care also inform individualized risk–benefit discussions.

## Methods

Cases and additional clinical scenarios were selected collaboratively by the primary and corresponding author to illustrate the breadth of indications, medical and psychiatric complexity, and safety considerations encountered through PEACE.

The Institutional Review Board determined that this project did not constitute research involving human subjects. Both patients provided written consent for publication. Additional clinical scenarios were drawn from retrospective review of PEACE clinical experience and are presented in de-identified form using pseudonyms.

## Case 1

Mr. Z is a 41-year-old man with moderate alcohol use disorder (AUD), major depressive disorder in partial remission, and hypertension who sought consultation at the PEACE clinic to explore psilocybin as a treatment for AUD.

### Pre-Psilocybin Clinical Course

Approximately nine months before his initial PEACE consultation, Mr. Z was drinking 5–6 beers most nights and had four emergency department visits over two months related to alcohol intoxication and suicidal ideation. He described drinking not “to get drunk,” but rather “to turn my brain off.” During this period, he experienced a near-fatal fentanyl overdose requiring resuscitation, despite no history of recreational opioid use, and a separate hanging attempt interrupted by family and law enforcement. Although he denied suicidal intent related to the overdose, it occurred amid severe depression, alcohol intoxication, and recurrent suicidal ideation. These events led to two brief inpatient admissions for detoxification and stabilization and an eight-week intensive outpatient program (IOP) for AUD. After completing the IOP, Mr. Z returned to full-time work. Within a month, however, he resumed alcohol use, averaging 2–6 drinks per week over the six months preceding his PEACE consultation.

## PEACE Consultation and Risk Assessment

At presentation to PEACE, Mr. Z remained on naltrexone and engaged in regular counseling, though he felt both were providing diminishing benefit. Despite substantially reduced alcohol consumption, he reported persistent cravings, preoccupation with alcohol, and difficulty “quieting the mind” without drinking.

His depressive symptoms were in partial remission with duloxetine 90 mg daily, bupropion XL 300 mg daily, and trazodone 50 mg nightly. His hypertension was well-controlled with lisinopril 20 mg daily. A prior therapist-documented diagnosis of bipolar II disorder was evaluated and felt to be inaccurate. Trauma screening revealed childhood exposure to predatory behavior by an adult neighbor and harsh physical punishment by his parents. Assessment identified no absolute contraindications to psilocybin, though his suicidal behavior nine months earlier, concurrent duloxetine and trazodone use, and trauma history warranted careful consideration.

Mr. Z was counseled that limited evidence suggested duloxetine might attenuate psilocybin effects for weeks to months after discontinuation and that abrupt cessation could cause withdrawal or depressive relapse.^27,28^ We discussed uncertain serotonin toxicity risk from combining duloxetine, trazodone, and psilocybin, while noting that risk appeared low for an SNRI with psilocybin alone.^29^ Despite advice to coordinate changes with his physician, he abruptly discontinued duloxetine, bupropion, and naltrexone before his second PEACE consultation one week later, without significant discontinuation symptoms or depressive relapse.

During his second virtual PEACE visit, also attended by his wife, we reviewed his individualized risks. We discussed transient blood pressure elevation and advised him to continue lisinopril on the day of dosing. We also discussed the potential emergence of challenging or trauma-related material. Given his recent discontinuation of duloxetine, we advised that blunting effects could persist and recommended waiting at least 1–2 months before dosing. He also received guidance on preparation, intention setting, safer dosing, and post-experience integration.

## Supervised Psilocybin Experiences and Initial Integration

Two months later, Mr. Z underwent the first of two supervised psilocybin sessions at a licensed Oregon service center. The sessions were conducted two months apart. He received the maximum state-permitted dose of 50 mg of psilocybin analyte in a botanical extract during each session, with a facilitator-led preparation session preceding each. He described the experiences as “quite positive” and “relatively smooth,” reporting insights into the “automaticity” of his alcohol use and reduced mental tension around drinking in the weeks afterward. In a follow-up message, he wrote:

I’ve spent my whole life believing in the primacy of the intellect. I realized that the primacy of the intellect gave way to the tyranny of the intellect. The mushrooms reconnected my mind and my body, and my physical craving/obsession of the mind to drink lost much of its power…

He later described recognizing intellectualization as a maladaptive defense mechanism that reinforced and rationalized his alcohol use and felt the psilocybin experiences helped disrupt these fixed patterns.

Following the psilocybin sessions, Mr. Z continued weekly alcohol counseling for approximately two months, attended one integration session after each dose, and reported drinking approximately 1–3 drinks per week during this period. This represented a modest reduction in consumption accompanied by a greater subjective reduction in the “mental tension” and compulsive thoughts driving his drinking. He restarted bupropion and remained off duloxetine and naltrexone.

## Return to Alcohol Use, Expanded Integration, and Abstinence

Over the three to four months following his second psilocybin session, his alcohol use gradually increased, eventually reaching as many as 14 drinks per week. After disclosing this escalation to his PCP, he was encouraged to re-engage with alcohol counseling and integration therapy. Although he did not see an integration therapist, he resumed regular alcohol counseling and continued follow-up with his PCP, both of whom incorporated discussion of his psilocybin experiences into ongoing care. About a year after his first psilocybin session, he achieved abstinence, later recognizing that, for him, attempts at moderation perpetuated his preoccupation with alcohol and abstinence was more sustainable.

## Unsupervised Psilocybin Use and Subsequent Integration

Nine months into abstinence, Mr. Z independently undertook an unsupervised high-dose psilocybin experience without prior consultation with his PCP, therapist, or PEACE team. Following a difficult experience characterized by derealization, existential disorientation, and isolation, he disclosed the experience to his PCP and was referred back to PEACE for integration-focused support. Care incorporated meaning-making and reflective exploration of self-compassion, self-forgiveness, and shame-based narratives, alongside mindfulness practices and values-oriented reflection. At the time of writing, Mr. Z reported approximately 18 months of sustained recovery, with the exception of a single episode of alcohol use involving one beer.

## Case 2

Mrs. X is a 79-year-old woman with major depressive disorder (MDD), stable hypertension, and hyperlipidemia who sought PEACE consultation to explore psilocybin as an alternative to long-term antidepressant treatment. Although her depression was in sustained remission, she hoped to discontinue antidepressants because of medication-associated sexual dysfunction and constipation and because of concern that they might attenuate the subjective effects of psilocybin.

## Pre-Psilocybin Clinical Course

Mrs. X was first diagnosed with MDD after experiencing postpartum depression in her early 30s. She described a strong family history of severe depression and adverse childhood experiences, including emotional neglect and childhood trauma in a household marked by maternal depression and paternal alcohol use. She began antidepressant treatment in her 40s and underwent multiple medication trials across several classes that were generally effective but limited by adverse effects. Independent attempts to discontinue treatment resulted in severe depressive relapses and two psychiatric hospitalizations in her 40s and 50s.

## PEACE Consultation and Risk Assessment

Mrs. X’s depression was in full remission (PHQ-2 = 0) while taking duloxetine 60 mg daily and bupropion immediate-release 100 mg three times daily, a regimen she had maintained for 20 years before her PEACE consultation. No medical or psychiatric contraindications to psilocybin use were identified, and she was assessed as having low risk for psychosis and serotonin syndrome. Her history of severe depressive relapse following antidepressant discontinuation was recognized as a risk-enhancing factor, though she also had protective factors of longstanding stability, current remission, and absence of suicidal ideation.

Mrs. X initially inquired about microdosing and was advised that, despite positive anecdotal and observational reports, thus far the controlled evidence supporting microdosing for MDD remains limited, whereas clinical trials have demonstrated potential benefit from psychedelic doses administered with structured psychological support. She was educated about potential attenuation of psilocybin effects by duloxetine and the risk of depressive relapse with antidepressant discontinuation.^27,28^ A gradual 10-week duloxetine taper plan was provided for implementation with her PCP, with continuation of bupropion recommended to reduce the risk of discontinuing both antidepressants simultaneously. After reviewing the PEACE recommendations, her PCP advised establishing longitudinal psychiatric care, which she did before her first psilocybin experience.

## Supervised Psilocybin Experiences and Initial Response

Mrs. X completed her duloxetine taper with her PCP and outpatient psychiatric nurse practitioner nine weeks after PEACE consultation and underwent her first supervised psilocybin session three weeks later at a licensed service center. She initially received approximately 25 mg of psilocybin analyte in a botanical extract, followed by a supplemental dose of approximately 8 mg, for an estimated total of 33 mg. A second session involving 35 mg of psilocybin analyte in a botanical extract followed two months later. Both sessions produced substantial subjective psychedelic effects. She described her first experience as emotionally challenging, centered on grief and personal trauma within the broader context of family relationships and intergenerational suffering as a descendant of Holocaust survivors. Themes of forgiveness also emerged. She completed one integration session with her facilitator.

Between sessions, Mrs. X reported reduced anxiety and remained without depressive symptoms (PHQ-2 = 0). She discontinued bupropion before her second psilocybin session, which she described as joyful. Three weeks later, her outpatient psychiatric nurse practitioner documented continued absence of depressive symptoms and substantial improvements in energy and motivation. Mrs. X stated, “It’s unbelievable to feel this way,” and reported resolution of sexual dysfunction and constipation. Together, they elected to continue without antidepressants and transition psychiatric follow-up to an as-needed basis.

## Depressive Recurrence and Escalation of Care

Five months after her second psilocybin session, Mrs. X returned to PEACE in a moderate depressive episode (PHQ-9 = 12). Irritability and anger emerging approximately three months after the second psilocybin session had progressed to low mood and hopelessness. She had independently restarted bupropion immediate-release 100 mg three times daily about 10 days before returning to PEACE.

During early aftercare visits, Mrs. X expressed that she wished she had received more longitudinal support following her psilocybin sessions. Her symptoms subsequently worsened, with anxiety, intense anger, insomnia, and expressions of not wanting to live, although she denied suicidal intent or plan. The severity of her symptoms prompted consideration of hospitalization. PEACE transitioned from its usual consultation-based model to longitudinal treatment. A PEACE psychiatrist assumed responsibility for medication management and integration-focused care, while a licensed clinical social worker (LCSW) within the institution provided skills-based psychotherapy. Initial treatment prioritized stabilization before deeper integration of her psychedelic experiences.

Medication management included titration of lamotrigine to 200 mg daily and conversion of bupropion immediate-release 100 mg three times daily to extended-release 300 mg daily. Her acute symptoms stabilized, and hospitalization was avoided. She also joined an online women’s integration support group, which she found highly beneficial.

## Longitudinal Integration-Focused Care and Recovery

Mrs. X received care from the PEACE psychiatrist for over one year and concurrent psychotherapy with the institutional LCSW for approximately nine months before transitioning to an outside therapist. The psychiatrist used an integrative approach incorporating meaning-making, depth-oriented and psychodynamic exploration, mindfulness practices, and inner-child work. Treatment addressed intergenerational grief and trauma, childhood neglect and abuse, forgiveness, and her tendency to overidentify with negative emotional states while discounting states such as contentment or bliss. Music from her psilocybin experiences was incorporated into some visits to facilitate deeper exploration of her emotional experience during the dosing sessions. Self-nurturing practices involving nutrition, positive self-directed cognitions, creative activities, and gardening were encouraged.

The LCSW incorporated approaches informed by acceptance and commitment therapy (ACT), dialectical behavior therapy (DBT), and cognitive behavioral therapy (CBT) to strengthen self-worth, support values-based decisions toward a fulfilling life, and develop distress-tolerance and self-soothing skills. Both clinicians also addressed her tendency to rely heavily on her partner and care team during distress, working to strengthen her agency and confidence in her own coping capacity.

Her depressive episode ultimately resolved, and she remained in remission for several months before completing treatment with PEACE. At the end of treatment, she remained off duloxetine while continuing bupropion and lamotrigine. Reflecting on the process, she expressed gratitude for the experience and an emerging sense that it had been necessary to “become the new me.”

## Discussion

These cases highlight a clinical framework for supporting patients with complex medical and psychiatric histories considering or pursuing psilocybin through Oregon’s regulated service center model. The first case involved a patient with alcohol use disorder who underwent two supervised and one unsupervised psilocybin sessions and ultimately achieved sustained recovery following a gradual and nonlinear recovery process. The second involved a patient with major depressive disorder who experienced an initial antidepressant response following two psilocybin sessions, followed by a severe depressive relapse requiring longitudinal integration-focused psychiatric care. Together with the broader clinical scenarios described, these cases illustrate both the potential benefits of psilocybin and the challenges of applying an emerging evidence base to medically and psychiatrically complex patients in real-world settings.

## Differences Between Clinical Trial Populations and Real-World Contexts

A growing number of randomized controlled trials support the potential efficacy of psilocybin for conditions including treatment-resistant depression, cancer-related depression and anxiety, and alcohol use disorder, although findings for AUD have been mixed.^19–25^ However, the effectiveness and safety of psilocybin in broader real-world populations, particularly among individuals with medical and psychiatric comorbidities or other risk factors, remain incompletely characterized.^26^ Participants in modern psilocybin trials typically undergo extensive medical and psychological screening and are subject to strict eligibility criteria.^26^ Contemporary psilocybin trials have commonly excluded individuals with significant psychiatric, cardiovascular, neurologic, or other medical risk factors, as summarized in Tables 2 and 3.^18–26^ Accordingly, direct evidence regarding risk in these populations remains limited. For some potential adverse outcomes, available evidence derives largely from case reports and observational data rather than systematic prospective research.^30^ As state-regulated access expands, clinicians and psilocybin facilitators may increasingly be asked to counsel patients whose medical and psychiatric complexity extends beyond the populations represented in existing psilocybin clinical trials. Although expert consensus recommendations for the future integration of psilocybin into clinical care have begun to emerge, standardized guidance for clinicians advising patients about state-regulated psilocybin services remains limited.^12^

This evidence gap is particularly relevant in Oregon, where state rules identify only three responses that categorically preclude participation in an administration session: lithium use within the preceding 30 days, current thoughts of or desire to cause harm to oneself or others, and ever having been diagnosed with or treated for active psychosis.^31^ Consequently, individuals who would have been excluded from clinical trials may still be eligible for community-based psilocybin services. Although facilitators complete state-approved training and conduct required eligibility screening, facilitator licensure does not require a medical or mental health credential or advanced clinical training in complex comorbidities and polypharmacy.^32,33^ Psilocybin consultation models such as PEACE may therefore provide a complementary resource for interpreting uncertain evidence and applying it to an individual patient’s clinical circumstances.

Consultation also requires contextualizing the strength and limitations of available evidence. This need frequently arises in discussions of PTSD, given the high prevalence of PTSD and lifetime trauma exposure among patients evaluated through PEACE.^14^ Evidence supporting psilocybin specifically for PTSD remains preliminary,^34^ and is eclipsed by a larger body of evidence from randomized trials of MDMA-assisted therapy for PTSD.^35^ Therefore we suspect patients may be prone to misattributing the literature from MDMA-assisted therapy to psilocybin services which could affect their outcomes. Similar contextualization is needed for indications such as OCD and fibromyalgia, for which promising findings have largely emerged from small studies without parallel control groups.^36,37^ Clinical consultation can help patients interpret findings in light of study design, sample size, comparators, and applicability to their individual circumstances.

## Differences Between Oregon Psilocybin Services and Clinical Trial Models

Differences between clinical trials and community psilocybin services extend beyond patient selection. Modern clinical trials have generally evaluated psilocybin within highly structured research protocols incorporating extensive screening, preparation and post-dose support, monitored dosing sessions, and longitudinal follow-up.^18–25^ These studies are typically conducted with medical oversight and prespecified procedures for safety monitoring and adverse-event management.^12,18–25^ Replicating the full infrastructure of a clinical trial may be neither feasible nor necessary in community settings. However, for clients requiring additional clinical support, consultation models such as PEACE can provide individualized education, facilitate connection with mental health professionals when indicated, and can be billed to insurance to mitigate overall cost to patients.

The cases presented here underscore the importance of examining outcomes beyond the dosing session itself. Oregon rules require attempted follow-up within 72 hours but do not require an integration session or longer-term monitoring.^38^ Both patients described meaningful psilocybin experiences with early improvement in target symptoms, but their subsequent clinical courses included complexities that emerged several months after their service-center encounters. The first patient’s sustained recovery emerged through a prolonged process that included return to use, continued treatment engagement with his primary care physician and alcohol counselor, and an unsupervised psilocybin experience associated with significant psychological distress. The second experienced a severe depressive relapse after initial improvement and subsequently required extended psychiatric and psychotherapeutic support before achieving more sustained recovery. These cases cannot determine whether additional preparation, integration, or structured follow-up would have altered their trajectories. However, they illustrate that early improvement following psilocybin administration may not guarantee durable recovery and highlight the potential importance of longitudinal care and multidisciplinary involvement in the integration process after dosing individuals who have clinical complexity. Future research should both quantitatively and qualitatively examine longer-term outcomes and factors that predict sustained improvement, relapse, and adverse events following psilocybin use.

## The PEACE Model: Bridging Healthcare and Community-Based Psilocybin Services

The PEACE model was developed in part to bridge community-based psilocybin services and conventional healthcare through individualized assessment, education, risk reduction, longitudinal follow-up, and collaboration with patients' existing treatment teams. PEACE is not intended to replace psilocybin facilitators or integration therapists, nor does it serve as a gatekeeper for access to psilocybin services. Rather, it functions as a complementary clinical resource, particularly for patients with substantial medical or psychiatric complexity or those seeking guidance in areas where evidence remains uncertain.

A central goal of this psilocybin consultation model is to help patients navigate psilocybin use within the context of their broader medical and mental healthcare rather than in parallel to it. For example, although both patients discontinued duloxetine, their cases should not be interpreted as a general PEACE recommendation to discontinue SSRIs or SNRIs. Consultants discuss the tradeoffs of continuation versus tapering, including possible attenuation of psilocybin effects, discontinuation symptoms, and relapse risk. Recommendations are individualized, and patients are encouraged to discuss proposed medication changes with their prescribing clinicians before implementation. Such coordination may reduce fragmentation, support more informed decision-making, and allow emerging benefits, relapses, adverse effects, and changing clinical needs to be recognized and addressed over time.

Models like PEACE may also support condition-specific referral pathways connecting patients with preparation, integration, facilitation, and clinical resources appropriate to their needs. Individuals seeking psilocybin for cancer-related distress, alcohol use disorder, or treatment-resistant depression, for example, may benefit from networks of clinicians, therapists, and facilitators with relevant expertise. Future research should evaluate not only safety and clinical outcomes, but also whether psilocybin consultation improves informed decision-making, care coordination, engagement with preparation and integration supports, continuity of care, and other patient-centered outcomes.

## Conclusion

As psilocybin becomes increasingly available outside research settings, clinicians, even those outside states with legally available services, are likely to encounter patients seeking guidance regarding its use. The cases presented here illustrate the challenges of applying an emerging evidence base to medically and psychiatrically complex individuals while also demonstrating the potential importance of continuity beyond the dosing session. Psilocybin consultation models may offer one framework for supporting informed decision-making, reducing fragmentation, and integrating psilocybin-related care into patients' broader medical and mental healthcare.

## Figures and Tables

**Table 1:** Core Domains of the PEACE Assessment Visit

Domain	Components
**Goals and intentions**	Reasons for considering psilocybin, target concerns, treatment goals, and expected outcomes.
**Medical history**	Relevant medical diagnoses, symptoms, treatment history, and hospitalizations, with attention to cardiovascular, neurologic, and oncologic considerations.
**Psychiatric history**	Relevant psychiatric diagnoses, symptoms, treatment history, hospitalizations, suicidality, self-harm, psychosis, mania, and other clinically relevant conditions.
**Medications and supplements**	Current and previous medications, supplements, treatment responses, and adverse effects, with attention to serotonergic agents, lithium, antipsychotics, sedative-hypnotics, and stimulants.
**Substance use**	Current and previous substance use, substance use disorders, and prior experiences with psilocybin or other psychedelics.
**Trauma history**	Screening for physical, sexual, emotional, and medical trauma.
**Psychosocial context**	Living circumstances, relationships, social supports, employment, education, spirituality, religion, and contemplative practices.
**Family history**	Relevant psychiatric, neurologic, and cardiac history, with particular attention to bipolar and psychotic disorders.
**Objective findings**	Mental status examination and review of available diagnostic studies, including relevant laboratory results, electrocardiography, and imaging.
**Records and care-team review**	Relevant health records and input from involved medical and mental health clinicians.
**Evidence appraisal**	Strength and applicability of evidence regarding potential efficacy for the target condition and safety in the context of comorbidities, medications, and other clinical factors.
**Preliminary clinical synthesis**	Identification of principal safety concerns and uncertainties; separate qualitative medical and psychiatric risk ratings (low, moderate, or high); and priorities for education, medication guidance, care coordination, and risk reduction.

**Table 2:** Selected Psychiatric Conditions and Circumstances Affecting Eligibility in Modern Psilocybin Clinical Trials

Condition or clinical circumstance	Pattern across reviewed protocols
Personal history of a primary psychotic disorder	Consistently excluded
Personal history of bipolar spectrum disorder or affective psychosis	Consistently excluded
Family history of psychotic or bipolar spectrum disorders	Commonly excluded
Acute or clinically significant suicide risk, including serious suicidal behavior	Commonly excluded
Clinically significant personality disorder affecting safe participation	Commonly excluded
Active or severe substance use disorder other than the condition under study	Commonly excluded

*Note.* Categories represent a qualitative synthesis of eligibility criteria from eight selected representative modern psilocybin trials and their associated protocols.^18–25^ This table is not intended to be comprehensive, to constitute a systematic review, or to serve as a list of clinical contraindications.

**Table 3:** Selected Medical Conditions and Circumstances Affecting Eligibility in Modern Psilocybin Clinical Trials

Condition or clinical circumstance	Pattern across reviewed protocols
Pregnancy or breastfeeding	Consistently excluded
Uncontrolled hypertension	Consistently excluded
Clinically significant or unstable cardiac disease, such as recent myocardial infarction, decompensated heart failure, or severe valvular disease	Commonly excluded
Epilepsy or clinically significant seizure disorder	Commonly excluded
Clinically significant arrhythmia or prolonged QTc	Commonly excluded
Recent stroke, transient ischemic attack, or significant cerebrovascular disease	Commonly excluded
Severe hepatic disease	Commonly excluded
Insulin-dependent or poorly controlled diabetes	Commonly excluded
Severe renal disease	Excluded in some protocols
Primary brain tumor or intracranial mass lesion	Excluded in some protocols
Clinically significant cognitive impairment or major neurocognitive disorder	Excluded in some protocols

*Note.* Categories represent a qualitative synthesis of eligibility criteria from eight selected representative modern psilocybin trials and their associated protocols, supplemented by the medical exclusion-criteria framework described by Williams et al.^18–26^ This table is not intended to be comprehensive, to constitute a systematic review, or to serve as a list of clinical contraindications.
